# Internet-Delivered Early Interventions for Individuals Exposed to Traumatic Events: Systematic Review

**DOI:** 10.2196/jmir.9795

**Published:** 2018-11-14

**Authors:** Naomi Ennis, Iris Sijercic, Candice M Monson

**Affiliations:** 1 Department of Psychology Ryerson University Toronto, ON Canada

**Keywords:** psychological trauma, secondary prevention, trauma and stressor-related disorders, internet

## Abstract

**Background:**

Over 75% of individuals are exposed to a traumatic event, and a substantial minority goes on to experience mental health problems that can be chronic and pernicious in their lifetime. Early interventions show promise for preventing trauma following psychopathology; however, a face-to-face intervention can be costly, and there are many barriers to accessing this format of care.

**Objective:**

The aim of this study was to systematically review studies of internet-delivered early interventions for trauma-exposed individuals.

**Methods:**

A literature search was conducted in PsycINFO and PubMed for papers published between 1991 and 2017. Papers were included if the following criteria were met: (1) an internet-based intervention was described and applied to individuals exposed to a traumatic event; (2) the authors stated that the intervention was intended to be applied early following trauma exposure or as a preventive intervention; and (3) data on mental health symptoms at pre-and postintervention were described (regardless of whether these were primary outcomes). Methodological quality of included studies was assessed using the Downs and Black checklist.

**Results:**

The interventions in the 7 studies identified were categorized as *selected* (ie, delivered to an entire sample after trauma regardless of psychopathology symptoms) or *indicated* (ie, delivered to those endorsing some level of posttraumatic distress). Selected interventions did not produce significant symptom improvement compared with treatment-as-usual or no intervention control groups. However, indicated interventions yielded significant improvements over other active control conditions on mental health outcomes.

**Conclusions:**

Consistent with the notion that many experience natural recovery following trauma, results imply that indicated early internet-delivered interventions hold the most promise in future prevention efforts. More studies that use rigorous methods and clearly defined outcomes are needed to evaluate the efficacy of early internet-delivered interventions. Moreover, basic research on risk and resilience factors following trauma exposure is necessary to inform indicated internet-delivered interventions.

## Introduction

### Background

Approximately 75% of individuals are exposed to a traumatic stressor in their lifetime that involves exposure to actual or threatened death, serious injury, or sexual violence [1]. The types of traumatic exposures that are most commonly experienced include sexual assault, witnessing another person getting killed or badly injured, sudden unexpected death, and life-threatening motor vehicle accidents [1]. Following trauma, the majority of individuals will experience subclinical symptoms of distress that abate over time without intervention, considered *natural recovery* [2-4]. Although natural recovery is the most common trajectory following trauma exposure, a subset of trauma-exposed individuals experience significant distress and impairment that require intervention to facilitate recovery to healthy levels of functioning [1]. These individuals may be diagnosed with posttraumatic stress disorder (PTSD), major depressive disorder, generalized anxiety disorder, panic attacks, and health-risk behavior such as substance abuse [5-7]. These disorders can be chronic and pernicious but may be preventable if interventions are delivered early following trauma exposure [8]. The purpose of this paper was to systematically review early interventions delivered through the internet for individuals exposed to trauma.

Although natural recovery is expected for most trauma-exposed individuals, trauma experts recommend that mental health professionals should not wait to provide care until problems are chronic and purport the value of early preventive interventions [9]. The Institute of Medicine defines prevention as efforts to reduce the incidence of a disorder, as opposed to reducing the prevalence [10]. Within this definition, preventive interventions have been organized into 3 categories: universal, selected, and indicated [11]. Universal interventions are provided to all members of a population regardless of risk for developing a disorder, for example, interventions applied to an entire population before a traumatic event regardless of trauma exposure. Selected interventions are intended for those who exhibit risk factors for the disorder but show no signs or symptoms of the disorder, such as individuals exposed to a traumatic event who may or may not be experiencing symptoms of the disorder. Indicated interventions are provided to only those who have subthreshold symptoms of the disorder or a subclinical diagnosis, for example, those who screen positive as experiencing symptoms of distress following trauma [10].

Universal intervention delivered before trauma exposure has been argued to be infeasible and too costly. However, as compared with other mental health disorders that have a prodromal phase (eg, schizophrenia) or a waxing and waning course (eg, depression), disorders that have an onset subsequent to trauma exposure (eg, PTSD and acute stress disorder) have a clear onset, providing a unique window for selected or indicated prevention. Following a traumatic event, individuals may present to emergency rooms or be seen by health care providers, and these circumstances are opportune for the provision of intervention [12]. In light of these considerations, this paper focuses on selected and indicated interventions delivered to individuals already exposed to trauma.

Reviews of early interventions for trauma-exposed individuals demonstrate that efficacy varies across modalities. The literature consistently contraindicates psychological debriefing as an intervention following trauma [9,11,13]. Interventions based on cognitive and behavioral principles have been found to be valuable in the prevention of posttraumatic mental health problems [9,11,14]. Despite their potential value, Feldner et al [11] highlight that face-to-face preventive interventions can be intensive, time-consuming, and costly. Moreover, many individuals who experience distress following trauma do not receive intervention [15] due to perceived stigma, difficulty scheduling appointments, and lack of access to care (eg, due to living in a remote location, lack of transportation, lack of financial resources [6]). Given these considerations, the internet may be a valuable platform to deliver early intervention. The internet provides a medium to deliver interventions with a wide reach as websites can be accessed remotely at a low cost, and users can maintain anonymity. Such interventions may be particularly valuable in reducing the economic and psychological burden of natural disasters, terrorist attacks, or war because internet-delivered interventions can be delivered on a large scale and accessed by the entire affected communities.

### Objective

Research suggests that internet-based interventions are feasible [16] and efficacious in reducing PTSD and other psychopathologies (eg, anxiety disorders) that may follow from trauma [17,18]. However, to date no research has systematically reviewed the literature on early interventions for trauma-exposed individuals delivered via the internet. Given the potential value of such interventions for targeting a large number of people following trauma in a cost-effective way, an understanding of the available interventions and their efficacy in preventing or ameliorating posttraumatic distress is important. This study systematically reviewed internet-delivered interventions intended to be delivered acutely following trauma exposure, and the empirical data on mental health symptom change following these interventions.

## Methods

### Literature Search

A search was conducted according to the Preferred Reporting Items for Systematic Reviews and Meta-Analysis (PRISMA) statement [19]. The literature search was conducted in PsycINFO and PubMed, using the search terms “Trauma” OR “Posttraumatic Stress” OR “Posttraumatic Stress Disorder” or “Recent Trauma” OR “PTSD” and “Early Intervention” OR “Preventive Intervention” and “Online” OR “Web-based” OR “Internet-based therapy” OR “Internet-delivered.” Searches were limited to papers published after 1991 when the internet became available in North America. Reference lists of reviews and meta-analyses on early interventions [8,14] and internet-delivered interventions for PTSD [17] were also reviewed.

### Selection Criteria

Two reviewers (NE and IS) screened identified abstracts and titles to identify full-text studies. Due to the nascency of internet-delivered interventions, and to review all available early posttrauma interventions, inclusion criteria were broad and not limited to controlled trials. Papers were included if the following criteria were met: (1) an internet-based intervention was described and applied to individuals exposed to a traumatic event; (2) the authors stated that the intervention was intended to be applied early following trauma exposure or as a preventive intervention; and (3) data on mental health symptoms at pre- and postintervention were described (regardless of whether these were primary outcomes). Internet-based interventions were defined as interventions delivered online via a computer or mobile phone platform. The terms internet-based and Web-based were used synonymously to describe such interventions. Papers that described stepped care interventions whereby 1 aspect of a broader intervention involved an internet-based intervention were included [20].

Papers were excluded if (1) the intervention was delivered via the telephone or videoconferencing [21]; (2) an intervention was described and implemented, but empirical data on participants’ mental health symptoms pre- and postintervention were not reported (eg, protocols for randomized controlled trials [22] and studies that only describe feasibility data [23,24]); and (3) the authors did not explicitly state that the intervention was preventive or intended to be delivered early following trauma. Level of agreement between the 2 reviewers (NE and IS) was 100%.

### Methodological Quality of Included Studies

A total of 2 raters (NE, IS) evaluated the methodological quality of each included empirical study (ie, only studies examining empirical evidence for the intervention) using the Downs and Black Checklist [25]. This checklist was selected because it evaluates the quality of both randomized and nonrandomized trials, given that both were included in this review. The checklist assesses items under the following subscales: reporting, external validity, internal validity (bias and confounding), and power. A modified version of the power item was used [26]. With this modification, a study can achieve a total possible score of 28. The test-retest reliability (*r*=.88), inter-rater reliability (*r*=.75), and internal consistency (Kuder-Richardson formula 20=.89) of the checklist are good [25]. Higher scores indicate greater quality. In this study, inter-rater reliability ranged from 85% to 100% on each article reviewed. Raters discussed each discrepancy and achieved consensus for all discrepantly rated items.

### Data Extraction

After screening for relevance, full papers were examined. Data on the intervention, sampling, recruitment, methodology, and design were extracted from all included studies. Data on mental health outcomes at pre- and postintervention as well as longer-term follow-up, if available, were extracted (ie, feasibility data were not reviewed). Any measures of mental health symptoms (eg, depression, anxiety, and worry) were reviewed, given the range of psychological responses that individuals might experience after trauma. Results from intent-to-treat analyses and completer analyses were extracted. Interventions were coded as “selected” or “indicated.” Selected interventions were defined as interventions that were delivered to the entire sample, regardless of whether or not they endorsed mental health symptoms. Interventions were coded as indicated when delivered only to participants endorsing cut-off criteria of mental health symptoms.

## Results

**Figure 1:**
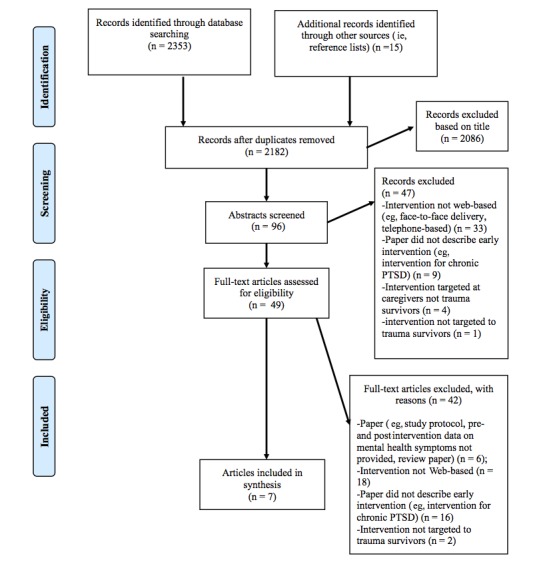
Flow diagram of literature search. PTSD: posttraumatic stress disorder.

Database searches yielded a total of 2346 articles. Review of reference lists of relevant articles yielded an additional 15 papers. Abstracts and titles were screened for inclusion. A total of 7 articles were included in the review based on selection criteria (see Figure 1). The most common reason for exclusion was that the intervention was not delivered via the internet or not an early or preventive intervention (eg, intended for chronic PTSD).

### Interventions

Descriptions of interventions are provided in Multimedia Appendix 1. Each paper described a different intervention. With regard to age of target population, 4 interventions were designed for adults [20,27-29], 1 for adults and adolescents [30], and 2 for children [31,32]. In terms of the type of trauma that interventions were designed to address, 4 interventions were intended for survivors of physical injury or medical events [20,27,31,32], 2 for individuals exposed to natural disasters [28,30], and 1 for veterans following combat [29]. No interventions specifically targeted survivors of interpersonal trauma such as sexual assault.

Each of the 7 interventions described were based on cognitive and behavioral principles. Moreover, 3 interventions were completely self-guided and consisted only of self-help psychoeducational materials [28,30,31]. Of the interventions, 1 intervention [32] was formatted as an interactive game with a storyline whereby children chose characters exposed to different types of trauma. The goal of the game was for users to help people in a town whose emotions had been *zapped*.

One intervention [20] was a stepped care approach in which patients were given laptops at their hospital bedside with access to an online community forum website, and they also met clinicians who consulted them about their intervention preferences. In the intervention described by Van Voorhees et al [29], patients received instant messages from clinicians and peers trained as counselors to encourage continued use of the website. The authors described this approach as motivational interviewing instant messages. The intervention described in Mouthaan et al [27] also had a peer support forum.

### Summary of Research Findings

The included studies are described in Table 1 and Multimedia Appendix 2. The quality of the studies according to the modified Downs and Black Checklist [26] ranged from 18 to 24, considered fair to good quality, with a median score of 21 (see Multimedia Appendix 1). Across all studies, assessors were not blind to patient intervention conditions. In only 1 study [29], adverse events that may have been important during the trial were reported, and only 2 studies [27,31] were adequately powered to detect a clinically important effect. Only 1 study [27] assessed symptom outcomes using gold standard clinician-administered measures and blinded assessment. There were 15 different assessment measures used across the studies because studies included multiple outcome measures. There were 3 assessment measures that were used across more than 1 study, and in each case, these measures were used in 2 studies only (see Table 1).

Across studies, participants were recruited from hospital emergency departments, intensive care units or surgical wards [20,27,31,32], random digit-dial methods in disaster-affected areas [30], online advertisements [29], and outpatient clinics [28]. There was variability with respect to the time the participants were recruited following trauma as well as the length of time that participants were followed after the intervention (Multimedia Appendix 2).

With regard to outcomes, in controlled studies of selected interventions (ie, all trauma-exposed participants received the intervention regardless of mental health symptoms), the interventions were not better than control conditions in reducing mental health symptoms. Means of outcome measures for intervention and control groups are reported in Multimedia Appendix 3. There was 1 exception to this finding. Ruggiero et al [30] found marginally statistically significant decreases in PTSD and depression symptoms in the group that received the *Bounce Back Now* intervention as compared with the assessment-only control group at 12-months postbaseline.

**Table 1:** Empirical data on internet-delivered early interventions.

Study (year)	Outcome measures	Intent-to-treat results	Completer results
Cox et al (2010) [31]	TSCC-A^a^; IES-R^b^	No advantage of intervention on any outcome at any assessment	Advantage of intervention on anxiety only, at both follow-up assessments
Kassam-Adams et al (2016) [32]	CPSS^c^; PedsQL^d^	No advantage of intervention on any outcome at any assessment	NR^e^
Mouthaan et al (2013) [27]	CAPS^f^; IES-R; HADS^g^	No advantage of intervention on any outcome at any assessment	Results similar to intention-to-treat (ITT) analyses for all outcome measures (statistics not reported in paper)
Ruggiero et al (2015) [30]	National Survey of Adolescents PTSD^h^, depression, substance use modules	No advantage of intervention on any outcome at any assessment	Results similar to ITT analyses for all outcome measures (statistics not reported in paper)
Steinmatz et al (2012) [28]	PSS^i^; CSE^j^; MPSS^k^; CES-D^l^; PSWQ^m^	Advantage of intervention on worry at postassessment.	NR
Van Voorhees et al (2012) [29]	CES-D 10; PCL-M^n^	Advantage of intervention on depression at 4 and 12 weeks and on PTSD at 4, 8, and 12 weeks	Advantage of intervention on depression at 4 and 12 weeks and PTSD at 4, 8, and 12 weeks
Zatzick et al (2015) [20]	PCL-C^o^; PHQ-9^p^	Advantage of intervention on PTSD at 6 months	NR

^a^TSCC-A: Trauma-Symptom Checklist for Children-A.

^b^IES-R: Impact of Event Scale-Revised.

^c^CPSS: The Child PTSD Symptom Scale.

^d^PedsQL: Pediatric Quality of Life Inventory.

^e^NR: not reported.

^f^CAPS: Clinician-Administered PTSD Scale.

^g^HADS: Hospital Anxiety and Depression Rating Scale.

^h^PTSD: posttraumatic stress disorder.

^i^PSS: Perceived Stress Scale.

^j^CSE: Coping Self-Efficacy Scale for Trauma.

^k^MPSS: Modified PTSD Symptoms Scale.

^l^CES-D: Center for Epidemiologic Studies Depression Scale.

^m^PSWQ: Penn State Worry Questionnaire.

^n^PCL-M: PTSD-Checklist Military version.

^o^PCL-C: PTSD Checklist Civilian version.

^p^PHQ-9: Patient Health Questionnaire-9.

The Bounce Back Now intervention is a combination of selected and indicated prevention wherein all participants had experienced trauma and were enrolled regardless of symptom presentation. However, within the intervention, modules were indicated based on participants’ symptomatology (eg, participants with a clinical level of symptoms of depression were invited and encouraged to use the depression module).

In the 3 studies in which interventions were indicated [20,28,29], significant reductions on some mental health symptoms were found compared with control conditions. Steinmatz et al [28] found that the group receiving the *My Disaster Recovery* intervention endorsed significantly greater reduction in worry over time as compared with the groups receiving online information only and the intervention-as-usual group. No significant differences among intervention conditions were found for symptoms of depression, PTSD, perceived stress, or coping self-efficacy. Van Voorhees et al’s [29] study did not have a control group, and they found significant decreases in depression and PTSD over time. Zatzick et al [20] found significant reductions in PTSD symptoms among participants in the intervention group from baseline to 6-month follow-up as compared with their usual care control. They did not find clinically or statistically significant differences between groups in depression scores over time. Although the intervention was delivered to all those who were exposed to trauma, regardless of symptom presentation, Kassam-Adams et al [32] conducted exploratory analyses separating those participants considered *at-risk* for posttraumatic stress (as indicated by baseline scores of 15 or higher on the Child PTSD Symptom Scale; [33]) from those *not-at-risk*. They found medium to large between-intervention-group effect sizes from baseline to 6 weeks (*d*=−0.84) and for baseline to 12 weeks (*d*=−0.68) for those at-risk, in favor of the intervention group. Small effect sizes were found for those not-at-risk between baseline to 6 weeks (*d*=−0.15) and for baseline to 12 weeks (*d*=−0.24).

## Discussion

### Principal Findings

The aim of this paper was to systematically review the literature on studies of internet-delivered early interventions for trauma-exposed individuals. Although previous reviews have identified numerous internet-delivered interventions for PTSD [17] and other chronic psychological disorders following trauma [16], this review identified only 7 studies that evaluated *early* internet-delivered interventions for trauma-exposed individuals. The lack of research on internet-based interventions following traumatization is interesting, given the potential low cost and wide-reaching impact of such prevention efforts.

### Overview of Included Studies

The quality of the included studies ranged from fair to good. Studies were generally not adequately powered to detect differences. In addition, most studies did not employ gold standard clinician-administered assessments and lacked long-term follow-up data. The objectives of studies varied as mental health symptoms were not the primary outcome of interest in several studies [28,32], and the same assessment measures were not used across studies. Mental health outcomes of interest also varied across studies (eg, PTSD, worry, and depression). The heterogeneity in outcomes assessed and general poor quality of assessment measures limit the conclusions that can be drawn about the effects of such interventions.

Despite the authors of these included studies describing the interventions as preventive or early interventions, the time since trauma varied widely across studies. For example, Van Voorhees et al’s [29] intervention was designed to be delivered acutely after return from deployment but was not delivered and tested until up to 5 years after deployment. The heterogeneity across studies in terms of when these interventions were provided is problematic. Interventions in these studies may not actually be *early* interventions, given the time elapsed since trauma, and therefore, findings may not represent the value of early interventions or be generalizable to individuals recently exposed to trauma. In addition, given that the trajectory of change in posttraumatic distress is most robust in the first 3 months following trauma exposure [3], it is recommended that early posttrauma intervention be tested within 3 to 6 months. Before conclusions can be drawn about the results of these studies, rigorous research is needed, and interventions should be delivered in the window of time after trauma within which the intervention was designed to be employed.

### Findings and Interpretation

Although limited conclusions can be drawn about the effects of the interventions because of the varied study quality and potential problems with timing of delivery in the included studies, a pattern emerged whereby interventions that were indicated (as compared with selected) tended to yield more promising results. In these studies, greater symptom improvement was found in intervention groups compared with controls. Interestingly, Kassam-Adams et al [32] provided the intervention to all trauma-exposed children and found no effect compared with the wait-list control. However, exploratory analyses showed larger effect sizes of the intervention for children at risk of PTSD compared with children not at risk. In studies of selected interventions, intervention groups faired similarly to control groups over time. This finding is in line with the notion that natural recovery is expected following trauma, and it suggests that the interventions may be most advantageous when delivered specifically to individuals demonstrating early symptoms of psychopathology. Delivering interventions when they are not indicated may not be cost effective since recovery without intervention is expected for the majority of those exposed to trauma.

Although data from this review and theory support that indicated interventions may be superior to selected early interventions, the potential for sampling bias across these types of interventions should be considered. For example, in over half of the included studies, the proportion of those who were approached to participate compared with the number who agreed was not reported, and no information was provided on how participating individuals might have differed from those who declined to participate. In indicated interventions, participants present with mental health symptoms. Individuals who self-select for a study and agree to participate may have more awareness of their symptoms and potentially more motivation for intervention, and this may contribute to better outcomes than studies of selected interventions. It is possible that stigma-related biases might render trauma-exposed individuals more likely to agree to participate in selected interventions where all individuals are given the intervention, regardless of whether they demonstrate natural recovery. Sampling bias must be considered when comparing outcomes across selected and indicated interventions, and reported in future studies.

Underscoring the importance of indicated over selected intervention, Feldner et al [11] hold that, given the high frequency of trauma exposure in the population, interventions should be delivered to those most at risk. Data on moderators of the interventions can contribute to better understanding of who will benefit the most from these interventions. However, none of the included studies examined moderators of intervention outcomes. In addition, potential mechanisms of interventions were not examined in any of the included studies, and thus, conclusions about the specific potency of specific strategies in these programs are limited.

Although the current data point to the potential benefits of indicated interventions, finding out whether users find them acceptable and sustainable and how far-reaching the interventions were is arguably as important as it is to ascertain whether the interventions are effective (ie, what communities they could reach). Data provided on accessibility, sustainability, and reach differed across studies when reported, with the majority not demonstrating far reach, given that the design was a pilot study. For example, Kassam-Adams et al [32] found that 35 of the 36 participants (participants were recruited from a hospital setting) accessed the website and roughly half completed the intervention. In contrast, in Ruggiero et al’s study [30] in which 2000 families were approached to participate in the intervention (via random digit dialing), approximately half of those approached accessed the website, and 37.5% completed one module. Just over a quarter of those who completed a module, completed the entire intervention. The differences in reach between studies and findings related to sustained use warrant further exploration.

### Limitations and Future Directions

Findings highlight several areas for future investigation. More research is needed before conclusions can be drawn about the efficacy and cost-benefit analysis of selected versus indicated prevention. The most common methodological weaknesses across the studies included variability in timing of the intervention (ie, interventions posited to be early interventions were not delivered acutely following trauma), lack of adequate power to detect significant differences, and potential sampling bias. To address design weaknesses in the current literature, rigorous and adequately powered research with clearly defined objectives should be conducted on existing interventions that utilize gold standard, blinded-clinician assessment, control groups, and follow-up data. Blinded-clinician assessment reduces potential bias, and clearly defined objectives may increase internal validity of the study.

Interventions should be delivered and tested in the intended acute phase after trauma. Accuracy in the timing of intervention delivery will ensure validity and generalizability of results. Due to the potential value of indicated over selected prevention, more research on posttrauma risk and resilience factors is necessary to determine for whom and how to target interventions. Participants should be recruited from diverse settings to determine the generalizability and efficacy of such interventions across survivors of different types of trauma. Moreover, to address potential sampling bias, recruiting participants from settings that draw trauma survivors for reasons other than mental health purposes (eg, motor vehicle accident reporting centers, emergency departments, or family physician offices) could be employed. By recruiting individuals who do not necessarily present for mental health posttrauma care, samples may include individuals who do not self-select based on predetermined symptoms of trauma. Future research should also identify barriers to accessing internet-delivered interventions.

In addition to lack of rigor across studies, the literature is limited in that none of the studies evaluated early internet-delivered intervention in survivors of interpersonal trauma (eg, sexual assault) specifically. Interpersonal trauma may be especially important to target, given its prevalence [34] and that most cases of posttrauma pathology stems from interpersonal trauma [35]. Online interventions aimed at victims of interpersonal trauma may be valuable in these populations and increase intervention seeking, because victims may be at risk for experiencing shame and stigma after trauma that could hinder them from seeking face-to-face mental health care [36].

Despite calls for interpersonal-based early posttrauma interventions [11] and findings that lack of posttraumatic social support is a potent risk factor for psychopathology [37,38], none of the interventions reviewed were interpersonally based (although 3 included peer support [20,27,29]). As none of the included studies examined mediators of intervention outcomes, little is known about whether peer support groups offered unique benefits compared with other aspects of the interventions. No studies examined the use of early interventions for individuals with poor posttraumatic social support, despite consistent findings that these individuals are at greater risk of posttraumatic pathology [37,38]. In studies where interventions were delivered to only those at-risk, risk was not defined in terms of social support (ie, always as symptom elevation). Feldner et al [11] hold that preventive interventions that mobilize social support may be best suited to naturalistic settings (eg, schools, religious communities), but the internet may serve as a valuable platform through which connections between socially isolated trauma survivors can be fostered. Researchers should continue to investigate interpersonal risk factors for posttraumatic psychopathology to develop and target such interventions.

In addition, although there was an approximately equal distribution of interventions included in this review that were targeted at adults, compared with children and adolescents, only 7 studies were reviewed. Researchers should continue to develop and study early posttraumatic interventions targeted at different age groups, given the prevalence of trauma across the life span [1]. There were also differences in terms of the complexity of the interventions delivered across the included studies, as some interventions [20,29,30] employed hybrid approaches (ie, stepped care, combination of a support group and clinician messages, self-help for parents in addition to an online intervention). Hybrid approaches may require more resources and could be more expensive [39]. However, there is a potential that such interventions may yield better results in alleviating psychopathology [39]. Researchers should continue to explore hybrid interventions compared with single treatments to determine the most parsimonious, cost-effective way to effectively prevent posttrauma psychopathology.

### Conclusions

To conclude, data suggest the potential efficacy of *indicated* early internet-delivered interventions in reducing mental health symptoms among trauma-exposed individuals experiencing elevated mental health symptoms. However, more high-quality, adequately powered studies are necessary before concrete conclusions can be drawn about the efficacy of such interventions.

## Appendix

Multimedia Appendix 1 Description of interventions in included studies.

Multimedia Appendix 2 Information on internet-delivered early interventions.

Multimedia Appendix 3 Mean scores on outcome measures at pre- and postintervention.

## Abbreviations

CAPS: Clinician-Administered PTSD Scale
CES-D: Center for Epidemiologic Studies Depression Scale
CPSS: The Child PTSD Symptom Scale
CSE: Coping Self-Efficacy Scale for Trauma
HADS: Hospital Anxiety and Depression Rating Scale
IES-R: Impact of Event Scale-Revised
ITT: intention-to-treat
MPSS: Modified PTSD Symptoms Scale
NR: not reported
PCL-C: PTSD Checklist Civilian version
PCL-M: PTSD-Checklist Military version
PedsQL: Pediatric Quality of Life Inventory
PHQ-9: Patient Health Questionnaire-9
PRISMA: Preferred Reporting Items for Systematic Reviews and Meta-Analysis
PSS: Perceived Stress Scale
PSWQ: Penn State Worry Questionnaire
PTSD: posttraumatic stress disorder
TSCC-A: Trauma-Symptom Checklist for Children-A

## References

[1] Van Ameringen M, Mancini C, Patterson B, Boyle MH (2008). Post-traumatic stress disorder in Canada. CNS Neurosci Ther.

[2] Ehlers A, Mayou RA, Bryant B (1998). Psychological predictors of chronic posttraumatic stress disorder after motor vehicle accidents. J Abnorm Psychol.

[3] Riggs DS, Rothbaum BO, Foa EB (2016). A prospective examination of symptoms of posttraumatic stress disorder in victims of nonsexual assault. J Interpers Violence.

[4] Rothbaum BO, Foa EB, Riggs DS, Murdock T, Walsh W (1992). A prospective examination of post-traumatic stress disorder in rape victims. J Trauma Stress.

[5] Galea S, Ahern J, Resnick H, Kilpatrick D, Bucuvalas M, Gold J, Vlahov D (2002). Psychological sequelae of the September 11 terrorist attacks in New York City. N Engl J Med.

[6] Hoge CW, Castro CA, Messer SC, McGurk D, Cotting DI, Koffman RL (2004). Combat duty in Iraq and Afghanistan, mental health problems, and barriers to care. N Engl J Med.

[7] Kilpatrick DG, Ruggiero KJ, Acierno R, Saunders BE, Resnick HS, Best CL (2003). Violence and risk of PTSD, major depression, substance abuse/dependence, and comorbidity: results from the National Survey of Adolescents. J Consult Clin Psychol.

[8] Roberts NP, Kitchiner NJ, Kenardy J, Bisson JI (2009). Systematic review and meta-analysis of multiple-session early interventions following traumatic events. Am J Psychiatry.

[9] Litz BT, Gray MJ, Bryant RA, Adler AB (2002). Early intervention for trauma: current status and future directions. Clin Psychol Sci Prac.

[10] Mrazek PG, Haggerty RJ (1994). Reducing Risk for Mental Disorders: Frontiers for Preventive Intervention Research.

[11] Feldner MT, Monson CM, Friedman MJ (2007). A critical analysis of approaches to targeted PTSD prevention: current status and theoretically derived future directions. Behav Modif.

[12] Qi W, Gevonden M, Shalev A (2016). Prevention of post-traumatic stress disorder after trauma: current evidence and future directions. Curr Psychiatry Rep.

[13] Agorastos A, Marmar CR, Otte C (2011). Immediate and early behavioral interventions for the prevention of acute and posttraumatic stress disorder. Curr Opin Psychiatry.

[14] Kliem S, Kröger C (2013). Prevention of chronic PTSD with early cognitive behavioral therapy. A meta-analysis using mixed-effects modeling. Behav Res Ther.

[15] Elhai JD, Terry N, Frueh CB (2005). Health service use predictors among trauma survivors: a critical review. Psychol Serv.

[16] Amstadter AB, Broman-Fulks J, Zinzow H, Ruggiero KJ, Cercone J (2009). Internet-based interventions for traumatic stress-related mental health problems: a review and suggestion for future research. Clin Psychol Rev.

[17] Sijbrandij M, Kunovski I, Cuijpers P (2016). Effectiveness of internet-delivered cognitive behavioral therapy for posttraumatic stress disorder: a systematic review and meta-analysis. Depress Anxiety.

[18] Sloan DM, Gallagher MW, Feinstein BA, Lee DJ, Pruneau GM (2011). Efficacy of telehealth treatments for posttraumatic stress-related symptoms: a meta-analysis. Cogn Behav Ther.

[19] Moher D, Liberati A, Tetzlaff J, Altman DG (2009). Preferred reporting items for systematic reviews and meta-analyses: The PRISMA statement. PLoS Med.

[20] Zatzick D, O'Connor SS, Russo J, Wang J, Bush N, Love J, Peterson R, Ingraham L, Darnell D, Whiteside L, Van Eaton E (2015). Technology-enhanced stepped collaborative care targeting posttraumatic stress disorder and comorbidity after injury: a randomized controlled trial. J Trauma Stress.

[21] Todder D, Matar M, Kaplan Z (2007). Acute-phase trauma intervention using a videoconference link circumvents compromised access to expert trauma care. Telemed J E Health.

[22] Freedman SA, Dayan E, Kimelman YB, Weissman H, Eitan R (2015). Early intervention for preventing posttraumatic stress disorder: an Internet-based virtual reality treatment. Eur J Psychotraumatol.

[23] Price M, Gros DF, McCauley JL, Gros KS, Ruggiero KJ (2012). Nonuse and dropout attrition for a web-based mental health intervention delivered in a post-disaster context. Psychiatry.

[24] Ruggiero KJ, Resnick HS, Acierno R, Coffey SF, Carpenter MJ, Ruscio AM, Stephens RS, Kilpatrick DG, Stasiewicz PR, Roffman RA, Bucuvalas M, Galea S (2006). Internet-based intervention for mental health and substance use problems in disaster-affected populations: a pilot feasibility study. Behav Ther.

[25] Downs SH, Black N (1998). The feasibility of creating a checklist for the assessment of the methodological quality both of randomised and non-randomised studies of health care interventions. J Epidemiol Community Health.

[26] Trac MH, McArthur E, Jandoc R, Dixon SN, Nash DM, Hackam DG, Garg AX (2016). Macrolide antibiotics and the risk of ventricular arrhythmia in older adults. Can Med Assoc J.

[27] Mouthaan J, Sijbrandij M, de Vries GJ, Reitsma JB, van de Schoot R, Goslings JC, Luitse JSK, Bakker FC, Gersons BP, Olff M (2013). Internet-based early intervention to prevent posttraumatic stress disorder in injury patients: randomized controlled trial. J Med Internet Res.

[28] Steinmetz SE, Benight CC, Bishop SL, James LE (2012). My Disaster Recovery: a pilot randomized controlled trial of an Internet intervention. Anxiety Stress Coping.

[29] Van Voorhees BW, Gollan J, Fogel J (2012). Pilot study of internet-based early intervention for combat-related mental distress. J Rehabil Res Dev.

[30] Ruggiero KJ, Price M, Adams Z, Stauffacher K, McCauley J, Danielson CK, Knapp R, Hanson RF, Davidson TM, Amstadter AB, Carpenter MJ, Saunders BE, Kilpatrick DG, Resnick HS (2015). Web intervention for adolescents affected by disaster: population-based randomized controlled trial. J Am Acad Child Adolesc Psychiatry.

[31] Cox CM, Kenardy JA, Hendrikz JK (2010). A randomized controlled trial of a web-based early intervention for children and their parents following unintentional injury. J Pediatr Psychol.

[32] Kassam-Adams N, Marsac ML, Kohser KL, Kenardy J, March S, Winston FK (2016). Pilot randomized controlled trial of a novel web-based intervention to prevent posttraumatic stress in children following medical events. J Pediatr Psychol.

[33] Foa E, Johnson K, Feeny N, Treadwell K (2001). The child PTSD symptom scale: a preliminary examination of its psychometric properties. J Clin Child Psychol.

[34] Centers for Disease Control Prevention (2014). CDC.

[35] Luthra R, Abramovitz R, Greenberg R, Schoor A, Newcorn J, Schmeidler J, Levine P, Nomura Y, Chemtob CM (2009). Relationship between type of trauma exposure and posttraumatic stress disorder among urban children and adolescents. J Interpers Violence.

[36] Ullman SE, Brecklin LR (2002). Sexual assault history, PTSD, and mental health service seeking in a national sample of women. J Community Psychol.

[37] Brewin CR, Andrews B, Valentine JD (2000). Meta-analysis of risk factors for posttraumatic stress disorder in trauma-exposed adults. J Consult Clin Psychol.

[38] Ozer EJ, Best SR, Lipsey TL, Weiss DS (2008). Predictors of posttraumatic stress disorder and symptoms in adults: a meta-analysis. Psychol Bull.

[39] Ho FY, Yeung WF, Ng TH, Chan CS (2016). The efficacy and cost-effectiveness of stepped care prevention and treatment for depressive and/or anxiety disorders: a systematic review and meta-analysis. Sci Rep.

