# Supplementary material for: Internet-Delivered Early Interventions for Individuals Exposed to Traumatic Events: Systematic Review
Source: J Med Internet Res. 2018 Nov 14;20(11):e280. doi: 10.2196/jmir.9795 (PMC6300083; doi:10.2196/jmir.9795)
Supplement: Multimedia Appendix 1 [file jmir_v20i10e280_app1.pdf]

Table 1. Description of interventions in included studies.

Reference	Name of Intervention	Theoretical orientation of intervention; Delivery format (eg, psychoeducational, interactive)	Description of sessions/modules; Length of intervention	Target population	Timing to be delivered posttrauma	Therapist delivered, peer support or completely self-guided	Total Quality Rating Score
Cox et al, (2010)	Kids and Accidents	Cognitive and behavioural, resilience theory; Self-guided psychoeducational	Self-help psychoeducational information; NR[a]; NR	8-12 year-olds with a potentially traumatic medical event	Within 2 weeks	Self-guided	21
Kassam-Adams et al, (2016)	Coping Coach	CBT[b] principles; Interactive game with a storyline	Three interactive modules on feelings, avoidance, and appraisal; each module 20-30 minutes	School-age children exposed to recent trauma	Early posttrauma period	Self-guided	23

Mouthan et al, (2013)	Trauma TIPS	CBT Techniques; Brief-self guided psychoeducation	Six self-guided modules (introduction to program, assessment of acute anxiety and arousal, video explanation, text summary of coping tips, contact information for professional help for enduring symptoms; and Web forum for peer support); 30-minute duration total	Adults exposed to unintentional injury	Immediately	Self-guided and web forum for peer support	24
Ruggiero et al, (2015)	Bounce Back Now	CBT; Interactive modules	Seven interactive modules on posttraumatic stress, depressed mood, generalized anxiety, alcohol use, marijuana use, illicit drug use, and cigarette use; length varies.	Adults and adolescents with symptoms of distress posttrauma	Early post natural disaster	Self-guided	22

Steinmatz et al (2012)	My Disaster Recovery	Social Cognitive Theory; Interactive modules	Six interactive modules on seeking professional help, relaxation, social support, unhelpful ways of coping, self-talk, and trauma triggers and memories; NR	Adults exposed to natural disaster	Acutely following disaster	Self-guided	19
Van Voorhes et al (2012)	VETS PREVAIL	CBT; Self-guided lessons and instant messaging component	Online structured lessons, online peer-to-peer instant messaging and brief instant messages from a clinical social work sessions with weekly peer-to-peer and clinical support; Length of time varies	Combat veterans recently deployed	Early intervention	Peer-to-peer support via chats and brief weekly motivational interviewing instant messages from clinical social workers	18

Zatzick et al (2015)	Afterdeployment.org and LifeArmor	CBT principles; Self-guided psychoeducation	Online self-assessments, self management strategies, videos, and psychoeducation materials; median time of 2.25 hours per patient[c]	Adults exposed to intentional or unintentional injury	Acutely following injury	Stepped care approach. Participants were encouraged to use Afterdeployment.org and LifeArmor application (smartphone application with the same material as Afterdeployment.org). Patients also had access to a care manager with whom they discussed PTSD treatment preferences. Afterdepoloyment.org and LifeArmor include community forum but do not include therapist interaction.	22

[a]NR = Not reported.

[b]CBT = Cognitive behavioural therapy.

[c]This intervention included contact with care managers. Median time included time spent in both online interactive components and with care manager.
