# Supplementary material for: Internet-Delivered Early Interventions for Individuals Exposed to Traumatic Events: Systematic Review
Source: J Med Internet Res. 2018 Nov 14;20(11):e280. doi: 10.2196/jmir.9795 (PMC6300083; doi:10.2196/jmir.9795)
Supplement: Multimedia Appendix 2 [file jmir_v20i11e280_app2.pdf]

Multimedia Appendix 2. Information on internet-delivered early interventions.

Study	N (# male); Mean age (SD)	Time since trauma	Setting	Intervention condition(s)	Design; F/U[a] periods	Selected or indicated
Cox et al (2010)	85 (59); 10.90 (2.18)	Within 72 hours of injury	Pediatric surgical wards	1: Kids and accidents; 2: assessment-only	RCT[b]; 4-6 weeks, 6 M[c]	Selected
Kassam-Adams et al (2016)	72 (39); 9.7 (NR)	Within 2 weeks of medical event	Hospital intensive care unit, general pediatric, or surgical unit	1: Coping coach; 2: Wait-list condition	RCT; 6, 12, 18 weeks	Selected[d]
Mouthaan et al (2013)	300 (180); 43.8 (15.9)	72 hours post injury	Academic Medical Center and VU University Medical Center	1: Trauma TIPS; 2. TAU[e]	RCT; 3, 6, 12 M	Selected
Ruggiero et al (2015)	2000 (981); 14.5 (1.7)	Within 12 M after tornado	Adolescents in local communities affected by tornadoes	1. Bounce Back Now; 2. Bounce Back Now + 7-module adult self-help; 3. Assessment-only	Population-based RCT; 4, 12 M	Selected but within modules indicated
Steinmatz et al (2012)	56 (8 male); 43.06 (11.51)[f]	11 M post hurricane Ike	University of Texas Medical Branch	1. My Disaster Recovery; 2. Information only website; 3. TAU	Pilot RCT; 1 M	Indicated (scores of 5 on the TSQ[g] or 20 on the PSS[h] included)
Van Voorhees et al (2012)	49 (44 male); 29.57 (7.17)	5 years post deployment	Social media, Prevail Health Solutions Website, Craigslist	1. VETS PREVAIL	Open trial; 4, 8, and 12 weeks	Indicated (subthreshold PTSD[i] and depression symptoms; 8-35 score on CES-D[j])
Zatzick et al (2015)	121 (78 male); 43.17 (14.69)	Immediately post injury	University of Washington's Harborview; Medical Center (trauma center, inpatient surgical ward, or emergency department)	1. Afterdeployment.org + LifeArmor Smartphone App 2. TAU	RCT; 1, 3, and 6 M	Indicated (scores of ≧35 on PCL-C[k] included)

[a]F/U: follow-up assessments.

[b]RCT: randomized controlled trial.

[c]M: months.

[d]Exploratory analyses were conducted comparing children with elevated posttraumatic stress disorder severity scores at baseline in both groups. Effect sizes were medium to large for baseline to 6 weeks (d=−0.84) and for baseline to 12 weeks (d=−0.68). Effect sizes were small for children not at risk from baseline to 6 weeks (d=−0.15) and for baseline to 12 weeks (d=−0.24).

[e]TAU: Treatment-As-Usual.

[f]Mean age (SD) for intervention group reported because mean age of entire sample not provided.

[e]NR: Not reported.

[g]TSQ: Trauma Screening Questionnaire.

[h]PSS: Perceived Stress Scale.

[i]PTSD: Posttraumatic stress disorder.

[j]CES-D: Center for Epidemiologic Studies Depression Scale.

[k]PCL-C: PTSD checklist civilian version.
