# Supplementary material for: Internet-Delivered Early Interventions for Individuals Exposed to Traumatic Events: Systematic Review
Source: J Med Internet Res. 2018 Nov 14;20(11):e280. doi: 10.2196/jmir.9795 (PMC6300083; doi:10.2196/jmir.9795)
Supplement: Multimedia Appendix 3 [file jmir_v20i11e280_app3.pdf]

Multimedia Appendix 3. Mean scores on outcome measures at pre- and postintervention.

Study	Outcome measures	Cut-off scores	Mean scores for intervention group	Mean scores for control group
Cox et al (2010)	TSCC-A[b]; IES-R[c]	NR[d]; 24=subclinical PTSD[e]; 33=probable PTSD dx[f]	TSCC-A Pre: 24.31 (13.73); TSCC-A Post: 18.90 (12.71); IES-R Pre: 10.72 (10.52); IES-R Post: 6.00 (7.50)	TSCC-A Pre: 18.07 (12.99); TSCC-A Post: 17.30 (16.85); IES-R Pre: 9.45 (9.53); IES-R Post: 5.11 (6.81)
Kassam-Adams et al (2016)	CPSS[g]; PedsQL[h]	≥12=probable PTSD dx; 78=special healthcare needs; 76=major chronic condition; 70=moderate condition	CPSS Pre: 18.4 (11.8); CPSS Post: 13.0 (12.1); PedsQL Pre: 78.0 (17.3); PedsQL Post: 75.1 (19.1)	CPSS Pre: 13.1 (7.3); CPSS Post: 12.5 (10.0); PedsQL Pre: 82.1 (15.8); PedsQL Post: 81.4 (15.1)
Mouthaan et al (2013)	CAPS[i;] IES-R; HADS[j]	≥45=PTSD dx; 24=subclinical PTSD; 33=probable PTSD dx; 8-10=mild; 11-14=moderate; 15-21=severe	CAPS Post: 17.7 (NR)[k]; IES-R Pre: 17.60 (16.82); IES-R Post: 10.6 (NR); HADS-anxiety Pre: 4.36 (3.90); HADS-anxiety Post: 4.6 (NR); HADS-depression Pre: 3.69 (3.50); HADS-depression Post: 3.6 (NR)	CAPS Post: 20.2 (NR); IES-R Pre: 21.22 (19.09); IES-R Post: 12.4 (NR); HADS-anxiety Pre: 4.87 (4.33); HADS-anxiety Post: 4.8 (NR); HADS-depression Pre: 4.13 (4.26); HADS-depression Post: 4.1 (NR)
Ruggiero et al (2015)	National Survey of Adolescents PTSD, depression, substance use modules	NR	BBN PTSD Pre: 2.73 (3.65); BBN PTSD Post: 1.34 (2.74); BBN+ASH PTSD Pre: 2.26 (3.25); BBN+ASH PTSD Post: 1.01 (2.15); BBN Depression Pre: 1.40 (2.00); BBN Depression Post: 0.79 (1.61); BBN+ASH Depression Pre: 1.22 (1.81); BBN+ASH Depression Post: 0.64 (1.30); BBN Alcohol Pre: 0.43 (3.03); BBN Alcohol Post: 0.78 (5.91); BBN+ASH Alcohol Pre: 0.67 (4.38); BBN+ASH Alcohol Post: 0.50 (4.56)	PTSD Pre: 2.54 (3.35); PTSD Post:1.30 (2.63); Depression Pre: 1.43 (1.96); Depression Post: 0.69 (1.40); Alcohol Pre: 1.12 (9.25); Alcohol Post: 0.92 (9.61)

Steinmatz et al (2012)	PSS[l]; CSE[m]; MPSS[n]; CES-D[o]; PSWQ[p]	0-13=low stress; 14-26=moderate stress; 27-40=high stress; N/A; ≥29=PTSD dx;≥20=clinical depression; 16-39=low worry; 40-59=moderate worry; 60-80=high worry	MDR PSS Pre: 21.06 (5.36); MDR PSS Post: 19.11 (7.14); IOW PSS Pre: 23.42 (6.09); IOW PSS Post: 23.58 (6.16); MDR CSE Pre: 104.11 (27.45); MDR CSE Post:109.44 (25.83); IOW CSE Pre: 93.00 (26.80); IOW CSE Post:101.26 (28.35); MDR MPSS Pre: 26.72 (17.39); MDR MPSS Post: 23.94 (16.74); IOW MPSS Pre: 29.84 (18.42); IOW MPSS Post: 29.26 (19.46); MDR CES-D Pre: 24.22 (10.21); MDR CES-D Post: 17.72 (11.74); IOW CES-D Pre: 24.58 (12.56); IOW CES-D Post: 22.58 (12.76); MDR PSWQ Pre: 52.83 (14.81); MDR PSWQ Post: 47. 94 (5.47); IOW PSWQ Pre: 59.68 (15.47); IOW PSWQ Post: 58.00 (15.21)	PSS Pre: 22.00 (4.96); PSS Post: 21.79 (5.37); CSE Pre: 87.63 (21.74); CSE Post: 100.58 (22.52); MPSS Pre: 28.26 (18.42); MPSS Post: 21.58 (16.30); CES-D Pre: 22.37 (12.10); CES-D Post:21.05 (11.03); PSWQ Pre: 51.37 (15.25); PSWQ Post: 52.68 (15.14)
Van Voorhees et al (2012)	CES-D 10; PCL-M[q]	≥9=clinically sig[r] symptoms of depression; ≥30=clinically sig symptoms of posttraumatic stress	CES-D Pre: 8.9 (4.0); CES-D > 9 Pre: 40 (20.0); CES-D Post: 7.3 (4.2); CES-D > 9 Post: 38.0 (19.0); PCL-M Pre: 35.0 (10.8); PCL-M > 30 Pre: 64.0 (32.0); PCL-M Post: 32.0 (11.4); PCL-M > 30 Post: 56.0 (28.0);	
Zatzick et al (2015)	PCL-C[s]; PHQ-9[t]	≥35=PTSD dx; 5-9=mild depression; 10-14=moderate depression; 15-19=moderately severe depression; 20-27=severe depression	PCL-C Pre: 46.91 (10.16); PCL-C Post: 44.37 (NR); PHQ-9 Pre: 14.23 (5.68); PHQ-9 Post: 13.39 (NR)	PCL-C Pre: 47.66 (10.85) PCL-C Pre: 44.64 (NR); PHQ-9 Pre: 15.17 (5.04); PHQ-9 Post: 14.00 (NR)

[a]Findings are reported on intent-to-treat analyses when available and on earliest postintervention assessment time points

[b]TSCC-A: Trauma-Symptom Checklist for Children-A.

[c]IES-R: Impact of Event Scale-Revised.

[d]NR: Not reported.

[e]PTSD: Posttraumatic stress disorder.

[f]dx: Diagnosis.

[g]CPSS: The Child PTSD Symptom Scale

[h]PedsQL: Pediatric Quality of Life Inventory.

[i]CAPS: Clinician-Administered PTSD Scale.

[j]HADS: Hospital Anxiety and Depression Rating Scale.

[k]CAPS pre-intervention scores not reported.

[l]PSS: Perceived Stress Scale.

[m]CSE: The Coping Self-Efficacy Scale for Trauma.

[n]MPSS: Modified PTSD Symptoms Scale

[o]CES-D: The Center for Epidemiologic Studies Depression Scale.

[p]PSWQ: Penn State Worry Questionnaire.

[q]PCL-M: PTSD-Checklist Military version.

[r]sig: Significant.

[s]PCL-C: PTSD Checklist Civilian version.

[t]PHQ-9: Patient Health Questionnaire-9
